# Carfilzomib Based Treatment Strategies in the Management of Relapsed/Refractory Multiple Myeloma with Extramedullary Disease

**DOI:** 10.3390/cancers12041035

**Published:** 2020-04-23

**Authors:** Xiang Zhou, Patricia Flüchter, Katharina Nickel, Katharina Meckel, Janin Messerschmidt, David Böckle, Sebastian Knorz, Maximilian Johannes Steinhardt, Franziska Krummenast, Sophia Danhof, Hermann Einsele, K. Martin Kortüm, Leo Rasche

**Affiliations:** 1 Department of Internal Medicine II, University Hospital of Würzburg, D-97080 Würzburg, Germany; 2 Mildred Scheel Early Career Center, University Hospital of Würzburg, D-97080 Würzburg, Germany

**Keywords:** carfilzomib, extramedullary disease, multiple myeloma, relapse, refractory

## Abstract

Published experience with carfilzomib in patients with relapsed/refractory multiple myeloma (RRMM) and extramedullary disease (EMD) is still limited. The current study aimed to assess the efficacy and safety of carfilzomib containing therapy regimens in EMD. We retrospectively analyzed 45 patients with extramedullary RRMM treated with carfilzomib from June 2013 to September 2019. The median age at the start of carfilzomib was 64 (range 40–80) years. Twenty (44%) and 25 (56%) patients had paraosseous manifestation and EMD without adjacency to bone, respectively. The serological overall response rate (ORR) was 59%. Extramedullary response was evaluable in 33 patients, nine (27%) of them achieved partial remission (PR) (ORR = 27%). In 15 (33%) patients, we observed no extramedullary response despite serological response. The median progression-free survival (PFS) and overall survival (OS) were five (95% CI, 3.5–6.5) and ten (95% CI, 7.5–12.5) months, respectively. EMD without adjacency to bone was associated with a significantly inferior PFS (*p* = 0.004) and OS (*p* = 0.04) compared to paraosseous lesions. Carfilzomib based treatment strategies showed some efficacy in heavily pretreated patients with extramedullary RRMM but could not overcome the negative prognostic value of EMD. Due to the discrepancy between serological and extramedullary response, evaluation of extramedullary response using imaging is mandatory in these patients.

## 1. Introduction

In multiple myeloma (MM), tumor growth is usually strongly dependent on the bone marrow microenvironment [1]. However, extramedullary disease (EMD) has been reported to occur in 6%–37% of MM patients, and the frequency increases during the course of the disease. The incidence can be as high as 70% in autopsy studies [2,3,4]. In addition, the pathogenesis of EMD remains largely unclear. Upregulation of angiogenesis and adhesion related genes might be a mechanism in EMD development [5]. C-X-C-motif chemokine receptor 4 (CXCR4) expression and hypoxia have also been reported to play a potential role in EMD growth [6,7]. Moreover, high-risk cytogenetics are significantly enriched in MM with EMD [8,9,10,11,12], and studies consistently showed EMD to be associated with poor outcome [12,13,14,15,16]. To date, there is no established approach for treatment of relapsed/refractory multiple myeloma (RRMM) with EMD.

Earlier, we and others reported on intensive multi-agent therapies, such as VDT-PACE (bortezomib, dexamethasone, thalidomide, cisplatin, doxorubicin, cyclophosphamide, and etoposide) and Dexa-BEAM (dexamethasone, carmustine, etoposide, cytarabine, and melphalan), which have shown some efficacy [17,18]. Furthermore, allogeneic stem cell transplantation has been reported to be an option for EMD [19]. In the phase 2 SIRIUS trial, the overall response rate (ORR) of daratumumab monotherapy was only 21.4% (3/14) in patients with EMD vs. 30.4% (28/92) of patients without EMD [20]. Notably, none of the above-mentioned strategies led to long-term disease-free survival. Therefore, identifying novel treatment strategies for patients suffering from EMD still represents an urgent medical need. Unfortunately, patients with extramedullary RRMM have only limited access to treatments within clinical trials due to the common exclusion criteria, such as non-secretory disease, central nervous system (CNS) involvement, and poor performance status.

Carfilzomib is a new generation proteasome inhibitor (PI), which in contrast to bortezomib, irreversibly binds to the 20S subunit of the proteasome and has been approved by the US Food and Drug Administration (FDA) for the treatment of RRMM [21]. In the phase 3 ENDEAVOR trial, superior efficacy of carfilzomib compared to bortezomib was observed in patients with RRMM [22], and patients with high-risk cytogenetics benefited from the replacement of bortezomib with carfilzomib [23]. Moreover, the combination of carfilzomib with immunomodulatory drugs (IMiD) lenalidomide and pomalidomide or the monoclonal antibody daratumumab is highly effective in RRMM [24,25,26]. However, published experience with carfilzomib in patients with extramedullary RRMM is still limited. The current study aimed to analyze carfilzomib containing therapy regimens in EMD.

## 2. Methods

### 2.1. Patients

We performed a single-center retrospective analysis. Utilizing our electronic database, we identified patients with extramedullary RRMM treated with carfilzomib-based regimens from June 2013 to September 2019 at our institution. RRMM was defined according to the current consensus recommendations [27]. EMD included paraosseous lesions originating from bone and extramedullary plasmocytomas without direct bone contact. The diagnosis of EMD was based on the histology of tumor bulk or, if biopsy was not possible, on imaging using computed tomography (CT), diffusion weighted magnetic resonance imaging (MRI), or positron emission tomography (PET). We did not include plasma cell leukemia in this analysis. Patients with at least one of the following aberrations were considered as high-risk cytogenetics: del(17p), t(4;14), t(14;16), and t(14;20) [28,29,30]. We retrieved and investigated patients’ demographic characteristics at diagnosis of MM and at the start of carfilzomib-containing treatment, MM related data (time point of diagnosis, subtype, cytogenetics, prior therapy lines, response and survival outcome), EMD related data (localization, adjacency to bones and secretory activity), treatment and adverse events (AEs) during therapy.

### 2.2. Treatment, Response, and Outcome

Carfilzomib was given on day 1, 2, 8, 9, 15, and 16 as intravenous (IV) short infusion in a 28-day cycle. We started carfilzomib at a dose of 20 mg/m^2^ on day 1 and 2 of the first cycle, and the dose was increased to 27 mg/m^2^ on day 8 if tolerated. Doses of carfilzomib were escalated or reduced according to the treating physician’s discretion. In our study, carfilzomib was administered in combination with at least one additional drug, including dexamethasone, IMiD, alkylating agent, and monoclonal antibody, i.e., daratumumab and elotuzumab.

We analyzed ORR, clinical benefit rate (CBR), overall survival (OS) and progression-free survival (PFS) following the current criteria of the International Myeloma Working Group (IMWG) [31,32]. Adverse events (AE) during carfilzomib containing treatment were characterized according to the Common Terminology Criteria for Adverse Events (CTCAE) Version 4.0.

### 2.3. Statistical Analysis

Using descriptive statistics, we summarized patients’ characteristics as absolute number and percentage, and if not otherwise stated as median and range. The survival analysis was performed with Kaplan–Meier method, and log-rank test was used to compare the survival outcome between subgroups. We used Fisher’s exact test to evaluate the difference in response rate (ORR and CBR) between the subgroups. These analyses were performed with GraphPad Prism 5.0. A *p*-value less than 0.05 was considered as statistically significant. 

## 3. Results

### 3.1. Patients’ Characteristics

In total, we identified 45 patients with extramedullary RRMM that were treated with carfilzomib containing regimens. The majority of the patients (*n* = 33, 73%) were male, and the median age at the start of carfilzomib for EMD was 64 (range 40–80) years. At the initial diagnosis of MM, primary EMD with and without adjacency to bone was already present in 16 (35%) and 1 (2%) patients, respectively. At presentation, 22 patients (49%) had high-risk cytogenetics from bone marrow biopsy. At start of carfilzomib as salvage therapy for secondary EMD, 20 (44%) and 25 (56%) patients suffered from EMD with and without adjacency to bone, respectively. Monoclonal protein in serum was detectable in the majority of the patients (*n* = 42, 93%). Muscle, skin, and soft tissue manifestation was the most frequent EMD presentation in our cohort (*n* = 38, 84%). Spinal cord and paravertebral lesions were seen in 25 patients (56%). Twenty (44%), 13 (29%), 11 (24%), and 2 (4%) patients had lymph node manifestation, malignant pleural effusion, parenchymal organ involvement, and gastrointestinal tract lesions, respectively. Lactate dehydrogenase (LDH) was elevated in 22 (49%) patients at start of carfilzomib for EMD.

In our cohort, patients had been treated with a median of four (range 1–9) prior lines of therapy. Forty-three (96%) patients were exposed to bortezomib, and eight (18%) patients had received carfilzomib prior to secondary EMD. Lenalidomide, pomalidomide, and thalidomide had been given in 35 (78%), 22 (49%), and 10 (22%) patients, respectively. Eighteen (40%) and three (7%) were treated with monoclonal antibodies daratumumab and elotuzumab, respectively. Forty-four (98%) and seven (15%) patients underwent autologous and allogeneic stem cell transplant (SCT), respectively. Seventy-three percent of the patients (*n* = 33) were refractory to the last line of therapy. Patients’ characteristics are summarized in Table 1.

### 3.2. Treatment and Response to Therapy

Overall, carfilzomib was administered twice weekly, and patients received a median of three (range 1–18) cycles of carfilzomib. The maximal dose of carfilzomib ranged from 15 to 56 mg/m^2^, and the majority of patients (*n* = 25, 56%) received a maximal carfilzomib dose of 27 mg/m^2^ twice weekly. All the patients were treated with dexamethasone 20–40 mg qw. The treatment regimens are summarized in Table 2. Regimens and doses were modified according to the treating physician’s choice. 

We first analyzed the best serological response in the 42 patients with M protein and measurable disease. Overall, we observed a serological ORR of 59% with 19% (*n* = 8) very good partial remission (VGPR) and 40% (*n* = 17) partial remission (PR). Ten (24%) patients achieved stable disease (SD), resulting in a serological CBR of 83%. Seven (17%) patients experienced serological progression while being treated with carfilzomib. Imaging follow-up data were available in 33 patients to determine the best response of EMD. No patient with isolated skin lesions, which could have been evaluated without imaging, was included in our study. We observed an extramedullary ORR of 27% (*n* = 9) with 27% (*n* = 9) PR. Nine (27%) patients had SD at the EMD sites, and, therefore, the CBR in this group was 54%. In addition, extramedullary progression was observed in nine (27%) patients. In six (18%) patients, a mixed response of EMD lesions was observed, with one EMD lesion progressing under therapy but another lesion responding. Data of response to treatment are summarized in Table 2. Similarly, a high proportion (*n* = 15, 33%) of differential response between serological parameters and EMD lesions was observed. Taken together, our data demonstrated an acceptable ORR in this heavily pretreated group of patients, but, notably, no complete remission (CR) could be achieved in our cohort.

KRd (carfilzomib, lenalidomide, and dexamethasone) was the most frequently administered regimen in our cohort (*n* = 17). In this subgroup, we observed a serological ORR of 76% with 47% (*n* = 8) VGPR and 29% (*n* = 5) PR, and a serological CBR of 88% with 12% (*n* = 2) SD. Extramedullary response was available in 11 patients of which 45% (*n* = 5) and 9% (*n* = 1) patients achieved PR and SD, respectively. Two (18%) patients had PD on treatment, and a mixed response was observed in three (27%) patients.

### 3.3. Survival Analyses

**Figure 1:**
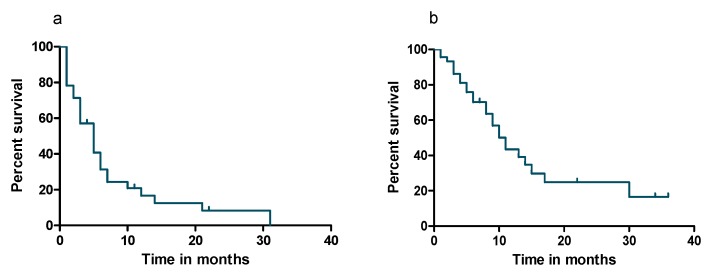
Progression-free survival (PFS) (**a**) (*n* = 45) and overall survival (OS) (**b**) (*n* = 45) of patients.

**Figure 2:**
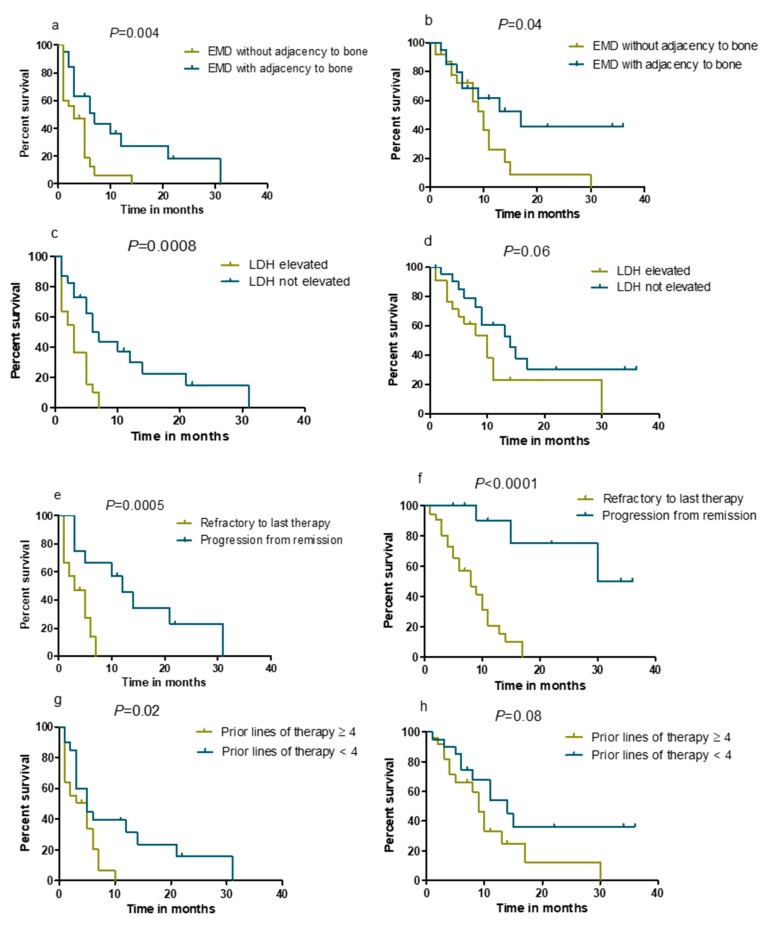
Progression-free survival (PFS) (**a**) and overall survival (OS) (**b**) in patients with extramedullary disease (EMD) adjacent to bone or not (EMD with adjacency to bone, *n* = 20; EMD without adjacency to bone, *n* = 25). PFS (**c**) and OS (**d**) in patients with normal vs. elevated lactate dehydrogenase (LDH) level (LDH < 250 IU/l, *n* = 23; LDH ≥ 250 IU/l, *n* = 22). PFS (**e**) and OS (**f**) in patients who were refractory to the last therapy line (*n* = 33) vs. who were not (*n* = 12). PFS (**g**) and OS (**h**) of patients with ≥ 4 prior therapy lines (*n* = 25) and < 4 prior therapy lines (*n* = 20).

In our cohort, median PFS and median OS were five (95% CI, 3.5–6.5) and ten (95% CI, 7.5–12.5) months, respectively (Figure 1a,b). In univariate analysis, patients suffering from extramedullary plasmacytomas without direct bone contact had a significantly inferior PFS (*p* = 0.004) and OS (*p* = 0.04) compared to those with paraosseous lesions only (Figure 2a,b). Furthermore, elevated LDH indicated a significantly inferior PFS (*p* = 0.0008), and a trend towards inferior OS (*p* = 0.06) (Figure 2c,d). Another negative prognostic factor was refractory disease to the last line of therapy with poorer PFS (*p* = 0.0005) and OS (*p* < 0.0001) compared to those with a progression from remission (Figure 2e,f). Furthermore, the PFS of patients who were treated with ≥ 4 prior lines of therapy was significantly shorter when compared with those who received less than four prior therapy lines (*p* = 0.02, Figure 2g). We also observed that patients who received ≥ 4 prior therapy lines had a trend towards an inferior OS compared to those treated with less than four prior lines of therapy (*p* = 0.08, Figure 2h). However, high-risk cytogenetics showed no significant negative prognostic value in our cohort (not shown in Figure 2). Due to the limited number of patients in our study, we did not perform a multivariate analysis.

### 3.4. Adverse Events (AEs)

Hematological AEs were the most common AEs during the treatment. We observed anemia, leukopenia, neutropenia, and thrombocytopenia grade ≥ 3 in 25 (56%), 24 (53%), 13 (29%), and 22 (49%) patients, respectively. When necessary, granulocyte colony-stimulating factor (G-CSF) and red cell or platelet concentrates were administered according to the current guidelines [33,34]. Among the non-hematological AEs, pneumonia (*n* = 6, 13%) was the most common AE grade ≥ 3. Additionally, five (11%) patients developed heart failure grade ≥ 3, possibly related to carfilzomib. In one of the five patients, the treatment had to be withdrawn after one cycle, and in the other four patients, treatment was continued after recovery of cardiac function. In total, two (4%) patients died due to pneumonia during the second and fourth cycles of treatment, respectively. One of both patients had a non-secretory MM with EMD and achieved an extramedullary mixed response after three cycles of Dara-KPd (daratumumab, carfilzomib, pomalidomide, and dexamethasone). In the other patient, PD was observed after one cycle of KRd. AEs are summarized in Table 3.

## 4. Discussion

The optimal management of extramedullary RRMM remains largely unclear. In the current study, we retrospectively analyzed the role of carfilzomib in the management of EMD. In total, we observed a serological ORR of 59% and a serological CBR of 83%. With regard to extramedullary response, ORR and CBR were 27% and 54%, respectively. To the best of our knowledge, there is no prospective study specifically investigating carfilzomib based treatment in EMD. Carfilzomib based treatments had shown EMD efficacy in case reports [35,36,37]. In a recent retrospective observational study, Muchtar et al. reported on a subgroup of patients with extramedullary RRMM who achieved a lower ORR (40%) and CBR (43.3%) when compared with patients without EMD (ORR = 49%, CBR = 63.5%) [38].

Of note, serological response differed from the extramedullary response in 33% of patients of our cohort, and six patients achieved an extramedullary mixed response. Indeed, intra-tumor genomic heterogeneity with advanced clones located to EMD might explain the lower response rates of these lesions compared to intramedullary disease [39,40]. Our study highlights the need for medical imaging in the follow-up of patients with EMD [41]. In our current study, the median PFS was five (95% CI, 3.5–6.5) months, and the median OS was ten (95% CI, 7.5–12.5) months. Comparably, Muchtar et al. reported a median duration of response of 3.9 months in patients with extramedullary RRMM, who were treated with carfilzomib containing regimens [38]. In a previous study of our institution investigating patients with EMD prior to the carfilzomib era, patients were treated with intensive chemotherapy, such as VDT-PACE, VRD-ICE, RAD, VCDT, and autologous or allogeneic SCT, and we observed a median PFS and OS of two (95% CI, 0.08–3.92) and seven (95% CI, 3.56–10.43) months, respectively [11]. Additionally, Rasche et al. reported a median PFS of four months in patients with extramedullary RRMM treated with DexaBEAM [17]. At our institution, the novel CXCR4-directed endoradiotherapy (ERT) has shown promising efficacy (ORR 75%) but no favorable survival outcome (median PFS 54 days, range 13–175 days and median OS 223 days, range 13–313 days) in extramedullary relapsed MM [42].

Our results suggest that patients with extramedullary plasmacytomas not adjacent to bone had a significantly inferior PFS and OS compared with those with paraosseous EMD. Similarly, Beksac et al. also reported that paraosseous lesions indicated a significantly superior PFS and OS when compared to extramedullary plasmacytomas without bone contact [43]. Furthermore, we observed that patients who were refractory to the last line of therapy represented a negative prognostic factor for PFS and OS. Our findings suggested that carfilzomib-containing treatment had only limited efficacy in this patient group.

Overall, five (11%) patients developed acute heart failure grade ≥ 3, and two (4%) patients died due to pneumonia during treatment. Hematological AEs including anemia, leukopenia, neutropenia, and thrombocytopenia grade ≥ 3 were observed in 25 (56%), 24 (53%), 13 (29%), and 22 (49%) patients, respectively. Due to the regular use of G-SCF and transfusion of blood products, these hematological AEs were well manageable. Generally, the rates of AEs grade ≥ 3 and the mortality during treatment were comparable to previous clinical studies investigating carfilzomib based therapy in RRMM [44,45,46].

There were several limitations to our current study. First, this is a retrospective analysis based on a relatively small number of patients who received heterogeneous carfilzomib containing treatment regimens. Second, the missing values might be a further limitation of our study.

## 5. Conclusions

In conclusion, compared to previous studies prior to the carfilzomib era, carfilzomib-based treatment strategies have shown similar ORR but improved survival outcome (PFS and OS) in patients with extramedullary RRMM. However, EMD still heralds poor prognosis, especially in patients being refractory to the last therapy line. In addition, due to the high proportion of discrepant response between serological parameters and EMD lesion, evaluation of extramedullary response using imaging should be performed in these patients.

## Figures and Tables

**Table 1:** Patients’ characteristics.

**Patients, *n***	45
**Gender, *n* (%)**	
Male	33 (73)
Female	12 (27)
**Subtype, *n* (%)**	
IgG	26 (58)
IgA	14 (31)
LC	5 (11)
**ISS Stage, *n* (%)**	
I	22 (49)
II	6 (13)
III	8 (18)
NA	9 (20)
**Cytogenetics, *n* (%)**	
High-risk	22 (49)
Standard-risk	18 (40)
NA	5 (11)
**Age at Start of Carfilzomib due to EMD Relapse, Median, Years (Range)**	64 (40–80)
**Bone Marrow Involvement at Start of Carfilzomib due to EMD Relapse**	
Yes	21 (47)
No	4 (9)
NA	20 (44)
**Serological MM Activity at Start of Carfilzomib due to EMD Relapse, *n* (%)**	
With Secretory Activity	42 (93)
Non-Secretory	3 (7)
**Serum LDH at Start of Carfilzomib due to EMD Relapse, *n* (%)**	
Elevated	22 (49)
Normal	23 (51)
**Prior Lines of Therapy, *n* (%)**	
1–2	15 (33)
3–5	16 (36)
≥ 6	14 (31)
**Response Status to The Last Therapy Line, *n* (%)**	
Refractory to The Last Line of Therapy	33 (73)
Progression from Remission	12 (27)
**Characteristics of EMD at Start of Carfilzomib, *n* (%)**	
EMD Adjacent to Bone	20 (44)
EMD without Adjacency to Bone	25 (56)
**Presentation/Localization of EMD, *n* (%)**	
Muscle, Skin, and Soft Tissue	38 (84)
Spinal Cord and Paravertebral Lesion	25 (56)
Lymph Node	20 (44)
Pleural Effusion	13 (29)
Parenchymal Organ	11 (24)
Gastrointestinal Tract	2 (4)
**Prior Treatment, *n* (%)**	
**PIs**	
Bortezomib	43 (96)
Carfilzomib	8 (18)
**IMiDs**	
Lenalidomide	35 (78)
Pomalidomide	22 (49)
Thalidomide	10 (22)
**Monoclonal Antibodies**	
Daratumumab	18 (40)
Elotuzumab	3 (7)
**SCT**	
Prior Autologous SCT	44 (98)
Prior Allogenic SCT	7 (15)

EMD—extramedullary disease; IMiDs—immunomodulatory drugs; ISS—The Multiple Myeloma International Staging System; LC—light chain; LDH—lactate dehydrogenase; MM—multiple myeloma; NA–not available; PIs—proteasome inhibitors; SCT—stem cell transplant.

**Table 2:** Treatment and response.

Pat	Regimen	Number of Cycles	Maximal Dose of Carf	Dosing of IMiD, Alkylating Agents and Monoclonal Antibodies	Best Response	
					Serology	EMD
1	Kd	11	56 mg/m^2^	N/A	PR	PR
2	Kd	1	15 mg/m^2^	N/A	SD	n.a.
3	Kd	1	56 mg/m^2^	N/A	PR	SD
4	KBd	1	27 mg/m^2^	Benda 70 mg/m^2^ qw	N/A	PD
5	KBd	1	27 mg/m^2^	Benda 70 mg/m^2^ qw	PD	PD
6	KCyd	5	27 mg/m^2^	Cyclo 200 mg qw	PR	SD
7	KCyd	2	56 mg/m^2^	Cyclo 300 mg qw	PR	n.a.
8	KCyd	1	27 mg/m^2^	Cyclo 750 mg qw	PR	PR
9	KCyd	1	20 mg/m^2^	Cyclo 300 mg qw	PR	mixed response
10	KCyd	3	27 mg/m^2^	Cyclo 300 mg qw	SD	SD
11	KRd	11	27 mg/m^2^	Rev 25 mg qd	PR	PR
12	KRd	7	20 mg/m^2^	Rev 15 mg qod	VGPR	PR
13	KRd	6	36 mg/m^2^	Rev 5 mg qd	VGPR	n.a.
14	KRd	2	20 mg/m^2^	Rev 5 mg qod	SD	n.a.
15	KRd	6	27 mg/m^2^	Rev 10 mg qd	VGPR	mixed response
16	KRd	3	27 mg/m^2^	Rev 10 mg qd	SD	PD
17	KRd	2	27 mg/m^2^	Rev 25 mg qd	PD	PD
18	KRd	2	27 mg/m^2^	Rev 25 mg qd	VGPR	n.a.
19	KRd	18	27 mg/m^2^	Rev 25 mg qd	VGPR	PR
20	KRd	6	27 mg/m^2^	Rev 25 mg qd	PR	mixed response
21	KRd	3	27 mg/m^2^	Rev 25 mg qd	PR	PR
22	KRd	5	27 mg/m^2^	Rev 25 mg qd	VGPR	PR
23	KRd	3	27 mg/m^2^	Rev 20 mg qd	PR	mixed response
24	KRd	9	27 mg/m^2^	Rev 25 mg qd	VGPR	n.a.
25	KRd	6	27 mg/m^2^	Rev 15 mg qd	PR	n.a.
26	KRd	3	27 mg/m^2^	Rev 10 mg qd	VGPR	SD
27	KRd	1	27 mg/m^2^	Rev 10 mg qd	PD	n.a.
28	KPd	3	27 mg/m^2^	Pom 4 mg qd	SD	mixed response
29	KTd	4	56 mg/m^2^	Thal 50 mg qd	SD	PD
30	KRCyd	1	20 mg/m^2^	Cyclo 300 mg qw, Rev 15 mg qd	PD	PD
31	KRCyd	3	56 mg/m^2^	Cyclo 300 mg qw, Rev 10 mg qd	PD	PD
32	KRCyd	3	27 mg/m^2^	Cyclo 300 mg qw, Rev 10 mg qd	SD	n.a.
33	KTCyd	4	36 mg/m^2^	Cyclo 300 mg qw, Thal 100 mg qd	PR	SD
34	Dara-Kd	2	56 mg/m^2^	Dara 16 mg/kg qw	PR	SD
35	Dara-Kd	1	20 mg/m^2^	Dara 16 mg/kg qw	PR	SD
36	Dara-KCyd	2	27 mg/m^2^	Dara 16 mg/kg qw, Cyclo 300 mg qw	SD	SD
37	Dara-KCyd	2	27 mg/m^2^	Dara 16 mg/kg qw, Cyclo 200 mg qw	N/A	n.a.
38	Dara-KCyd	3	15 mg/m^2^	Dara 16 mg/kg qw, Cyclo 200 mg qw	PR	PR
39	Dara-KCyd	5	56 mg/m^2^	Dara 16 mg/kg qw, Cyclo 300 mg qw	PR	SD
40	Dara-KPd	3	27 mg/m^2^	Dara 16 mg/kg qw, Pom 3 mg qd	N/A	mixed response
41	Dara-KPd	2	20 mg/m^2^	Dara 16 mg/kg qw, Pom 2 mg qod	PD	PD
42	Dara-KPCyd	1	20 mg/m^2^	Dara 16 mg/kg qw, Pom 2 mg qd, Cyclo 250 mg qw	PD	n.a.
43	Dara-KPCyd	4	27 mg/m^2^	Dara 16 mg/kg qw, Pom 2 mg qd, Cyclo 200 mg qw	SD	PD
44	Dara-KPCyd	1	15 mg/m^2^	Dara 16 mg/kg qw, Pom 2 mg qd, Cyclo 200 mg qw	SD	n.a.
45	Elo-KPd	1	36 mg/m^2^	Elo 10 mg/kg q2w, Pom 2 mg qd	PR	PR

Benda—bendamustine; Carf—carfilzomib; Dara—daratumumab; Dara-Kcyd—daratumumab, carfilzomib, cyclophosphamide, dexamethasone; Dara-Kd—daratumumab, carfilzomib, dexamethasone; Dara-KPCyd–daratumumab, carfilzomib, pomalidomide, cyclophosphamide, dexamethasone; Dara-KPd—daratumumab, carfilzomib, pomalidomide, dexamethasone; Elo—elotuzumab; Elo-KPd—elotuzumab, carfilzomib, pomalidomide, dexamethasone; EMDextramedullary disease; ImiD—immunomodulatory drugs; KBd—carfilzomib, bendamustine, dexamethasone; KcyD—carfilzomib, cyclophosphamide, dexamethasone; Kd—carfilzomib, dexamethasone; KPd—carfilzomib, pomalidomide, dexamethasone; KRCyd - carfilzomib, lenalidomide, cyclophosphamide, dexamethasone; KRd—carfilzomib, lenalidomide, dexamethasone; KTCyd—carfilzomib, thalidomide, cyclophosphamide, dexamethasone; KTd—carfilzomib, thalidomide, dexamethasone; N/A—not applicable; n.a.—not available; Pat—Patient; PD—progressive disease; Pom—pomalidomide; PR–partial remission; Rev—lenalidomide; SD–stable disease; Thal—thalidomide; VGPR—very good partial remission.

**Table 3:** Adverse events during therapy.

Adverse Events	Any Grade ≥ 2	Grade 3	Grade 4
**Hematological Events, *n* (%)**			
Anemia	37 (82)	25 (56)	
White Blood Cell Decreased	32 (71)	19 (42)	5 (11)
Neutrophil Count Decreased	30 (67)	7 (16)	6 (13)
Platelet Count Decreased	29 (64)	9 (20)	13 (29)
Febrile Neutropenia	1 (2)		1 (2)
**Non-Hematological Events, *n* (%)**			
Pneumonia	6 (13)	4 (9)	
Heart Failure	6 (13)	5 (11)	2 (4)
Influenza	4 (9)	4 (9)	
Upper Respiratory Infection	3 (7)	3 (7)	
Liver Enzyme Increased	2 (4)	1 (2)	
Urinary Tract Infection	2 (4)	2 (4)	
Cytokine Release Syndrome	1 (2)	1 (2)	
Gastrointestinal Infection	1 (2)	1 (2)	
Catheter Related Infection	1 (2)	1 (2)	
Peripheral Polyneuropathy	1 (2)	1 (2)	
Convulsion	1 (2)	1 (2)	
Renal Failure	1 (2)	1 (2)	
Oral Hemorrhage	1 (2)	1 (2)	
Bacterial Meningitis	1 (2)	1 (2)	
Skin Infection	1 (2)	1 (2)	
Sinusitis	1 (2)	1 (2)	
Atrial Fibrillation	1 (2)		
Thromboembolic Events	2 (4)		
Death	2 (4)		
